# Prevalence and predictors of early gestational weight gain associated with obesity risk in a diverse Australian antenatal population: a cross-sectional study

**DOI:** 10.1186/s12884-017-1482-6

**Published:** 2017-09-07

**Authors:** K. Cheney, S. Berkemeier, K.A. Sim, A. Gordon, K. Black

**Affiliations:** 1 Women’s and Babies, Royal Prince Alfred Hospital, Missenden Road, Camperdown, Sydney, NSW 2050 Australia; 2 Division of Obstetrics and Gynaecology, Campbelltown Hospital, Sydney, NSW 2560 Australia; 3 Discipline of Obstetrics, Gynaecology and Neonatology, The University of Sydney, Sydney, NSW 2006 Australia; 4 The Boden Institute, Charles Perkins Centre, The University of Sydney, Sydney, NSW 2006 Australia; 5 Charles Perkins Centre, The University of Sydney, Sydney, NSW 2006 Australia

**Keywords:** Early pregnancy, Obesity, Overweight, Gestational weight gain

## Abstract

**Background:**

Excess gestational weight gain (GWG) leads to adverse short- and long-term consequences for women and their offspring. Evidence suggests that excess GWG in early pregnancy may be particularly detrimental, contributing to the intergenerational cycle of obesity. The primary outcome was to investigate the prevalence and predictors of excess GWG in early pregnancy, and if women understand the risks to themselves and their offspring stratified by maternal body mass index (BMI).

**Methods:**

This was a secondary analysis (*n* = 2131) of a cross-sectional study (*n* = 2338) conducted over 6 months in 2015 of pregnant women attending antenatal clinics at four maternity hospitals across Sydney, Australia before 22 completed weeks gestation An self-completed questionnaire was used to investigate knowledge of expected weight gain in pregnancy, understanding of risks associated with excess GWG, self-reported anthropometric measures and socio-demographic data.

**Results:**

One third (34.2%) of women gained weight in excess of the recommendations by 22 completed weeks gestation. Women who were overweight (OR: 1.69, 95% CI: 1.33–2.14) or obese (OR: 1.64, 95% CI: 1.20–2.24) pre-pregnancy were more likely to gain excess weight in early pregnancy compared to normal weight women; as were women from lower socio-economic areas (OR: 1.89, 95% CI: 1.49–2.41). Half (51%) the women were unsure about the effect of excess GWG on their baby; 11% did not believe that excess GWG would affect the weight of the baby and 14% did not believe that excess GWG would affect longer term outcomes for their baby. Women who gained weight above the recommendations were significantly more likely to believe that excessive GWG in pregnancy would not have any adverse future effect on health outcomes or weight of their baby.

**Conclusions:**

The women at particular risk of excess early GWG are those who are overweight and obese and/or residing in lower socio-economic areas. These women need to be targeted for appropriate counselling preconception or in early pregnancy. Given the significant adverse outcomes associated with excess GWG in early pregnancy, preconception or early pregnancy counselling with respect to GWG and intervention research regarding best approach remains a public health priority.

**Electronic supplementary material:**

The online version of this article (10.1186/s12884-017-1482-6) contains supplementary material, which is available to authorized users.

## Background

Excessive gestational weight gain (GWG) is an independent risk factor for a myriad of short and long-term complications for both the mother and her offspring [1–3]. In 2009, the United States Institute of Medicine (IOM) published updated guidelines to provide direction to health professionals around healthy GWG expectations [4]. These guidelines are pre-pregnancy body mass index (BMI) specific and are used to guide clinicians when counselling women regarding weight gain in pregnancy.

While research has focused on overall GWG, trimester specific gains are of growing interest with respect to adverse outcomes. In the short term, excess GWG in the first half of pregnancy irrespective of pre-pregnancy BMI has been shown to increase the risk of gestational diabetes, hypertensive diseases of pregnancy and macrosomia [5–8]. Longer term consequences include greater postpartum weight retention, increased waist circumference and systolic blood pressure in women up to 7 years postpartum [9, 10]. Excess early pregnancy weight gain, in particular, has been shown to be an independent risk factor for development of gestational diabetes and maternal hyperglycaemia [6, 11, 12]. This may be due to the increase in maternal fat mass during early pregnancy which can increase insulin resistance and thus increase the risk of impaired glucose intolerance [11]. Furthermore, while there should be minimal gain physiologically in the first trimester, excess first trimester weight gain has also been shown to increase the risk of overweight and obesity in offspring in the first 4 years of life [13, 14].

A lack of awareness regarding the risk of excess GWG on health outcomes for the mother and her offspring may be an important factor for excessive GWG [15]. Pregnant woman who are already overweight or obese are less likely to be educated about weight gain recommendations than normal weight women, and have been shown to receive information conflicting with the IOM recommendations [16].

## Methods

The primary aim of this study was to examine the prevalence and specific health and socio-economic factors which may predict excess early GWG in a diverse early pregnant population. Secondly, we sought to examine the participants understanding of their GWG expectations specific to their BMI and, the significance of GWG on the health outcomes for their baby.

### Participants

This was a secondary analysis of a questionnaire (Additional file 1: Table S1) based study examining a relationship between BMI and pregnancy intention. At the second antenatal clinic visit, questionnaires were given to women who then returned them to a collection box. At the Royal Prince Alfred Hospital (RPAH) and Canterbury Hospital, a full-time midwife researcher recruited the women, while at Fairfield and Campbelltown Hospitals midwives in the antenatal clinic recruited on a pragmatic basis. Participants indicated consent by signing g a consent form and ticking a box on the questionnaire and completed it privately. Refusals were recorded at all of recruitment centers. This study was approved by the Human Research Ethics Committees of the participating sites. Ethics approval was obtained from Sydney Local Health District Ethics Review Committee (protocol number X14–0341; HREC/14/RPAH/459) and from the Sydney South West Area Health Service Ethics Review Committee (HREC/14/LPOOL/201).

### Study protocol

The original cross sectional analysis of 2313 women was conducted between March and December 2015, using an anonymous, self-completed brief questionnaire including health, socio-demographic data, anthropometry, a self-reported pre-pregnancy weight and weight measured at the second antenatal visit. All women were eligible to complete the survey if they were over 16 years of age, less than 22 weeks pregnant, had a singleton pregnancy and were able to complete the questionnaire in English or one of the other languages the questionnaire was translated into (Mandarin, Vietnamese, Arabic and Korean; the most common language groups at the participating hospitals). The cut-off gestation of 21^+6^ weeks was used as it was the gestation at the second antenatal visit and half way through the second trimester. Women specified their gestational age (by completed weeks) at the time of completion of the survey.

### Measurements

GWG was calculated by subtracting their self-reported pre-pregnancy weight from the maternal weight measured at the time of the second visit, allowing for the maximum recommended GWG in the first trimester weight gain of 2 kg [1] and then calculating the mean total weekly weight gain (in grams) by dividing their weight gain beyond the first trimester by the amount of weeks in their second trimester that were completed This was compared with the IOM recommended rate of GWG specific for each BMI category (Table 1). Excess weight gain was defined as greater than the recommended upper limit of weekly weight gain per the IOM guidelines for the specific pre-pregnancy BMI categories. BMI is calculated as weight in kilograms divided by height in metres squared (kg/m^2^).

**Table 1:** Institute of Medicine weight gain recommendations for pregnancy by BMI category [1]

Pre-pregnancy body mass index	Body mass index (kg/m^2^)	Recommended range of total weight (kg)	Recommended rates of weight gain ^a^ in the second and third trimesters (kg) (mean range [kg/week])
Underweight	< 18.5	12.5–18	0.51 (0.44–0.58)
Normal weight	18.5–24.9	11.5–16	0.42 (0.35–0.50)
Overweight	25–29.9	7–11.5	0.28 (0.23–0.33)
Obese (includes all classes)	≥ 30	5–9	0.22 (0.17–0.27)

^a^Calculations assume a 0.5–2 kg weight gain in the first trimester

GWG and BMI were also examined with respect to other sociodemographic and health determinants. Women were asked to specify their postcode in order to establish their socio-economic status (SES) using the index of relative disadvantage; a low score indicates a comparative lack of social advantage [17]. Ethnicity was self-reported and was categorised into ethnic groups; Caucasian, Asian (including Southern Asian), Middle Eastern/African, Pacific Islander/Maori, South American and Aboriginal/Torres Strait Islander. Women were also asked to complete questions regarding how much weight they expected to gain during the pregnancy and their understanding of the effect of excessive GWG on their pregnancy, the weight of their baby and their knowledge of longer term outcomes.

### Statistical analysis

Univariate and multivariable logistic regression was used to examine the association between excess maternal GWG, sociodemographic characteristics as well as expected GWG and understanding of GWG on outcomes. The variables included in the multivariable logistic regression model were those that had significance with a *p* value <0.25 in the univariate analysis as well as those considered relevant to the research question which was informed by the literature, for example, age group. Chi-squared significance tests were performed to establish whether there was an association with excess weight gain in the specific BMI categories, excess weight gain and understanding of the effect of weight gain on outcomes for the baby. Statistical analysis was performed using IBM© Statistical Package for the Social Sciences (SPSS) version 23.0 (Armonk, NY: IBM Corp).

## Results

A total of 2313 women completed the questionnaire, of these 2021 were included in this study. Those excluded (*n* = 292, 12.6%) had missing anthropometry data necessary to enable analysis of GWG or BMI. There were 14 refusals at RPA and Canterbury Hospitals (0.6%), 40 (15.7%) refusals at Campbelltown and 12 (0.3%) from Fairfield. The mean gestational age at recruitment was 19.4 weeks. Maternal socio-demographic characteristics and related variables are shown in Table 2. Underweight women comprised 6.4% (*n* = 129), normal weight 60.7% (*n* = 1227), overweight 21.1% (*n* = 427) and 11.8% (*n* = 238) obese.

**Table 2:** Frequency distribution of maternal sociodemographic characteristics

	Frequency (%)
Age (years)	
16–24 years	353 (17.1%)
25–34 years	1279 (61.9%)
≥ 35	424 (20.5%)
Maternal body mass index (kg/m^2^)	
Underweight (< 18.5)	129 (6.4%)
Normal weight (18.5–24.9)	1227 (60.7%)
Overweight (25–29.9)	427 (21.1%)
Obese (> 30)	238 (11.8%)
Relationship status	
Married/de-facto	1859 (89.9%)
Single/widowed/separated	192 (9.3%)
Smoking	
Non-smoker	1974 (95.6%)
Smoker	87 (4.2%)
Maternal ethnicity	
Caucasian	824 (40%)
Asian	815 (39.5%)
Middle Eastern/Africa	237 (11.5%)
Aboriginal or Torres Strait Islander	32 (1/6%)
Pacific Islander/Maori	85 (4.1%)
South American (Latino)	28 (1.4%)
Maternal religion	
Christian	735 (35.6%)
Hindu	143 (6.9%)
Buddhist	104 (5%)
Muslim	270 (13.1%)
No religion	724 (35%)
Other	91 (4.4%)
Maternal education	
Tertiary	1090 (52.7%)
High school	525 (25.4%)
Other (nil, primary)	412 (19.9%)
Socio-economic status	
Low	611 (29.6%)
Medium	243 (11.8%)
High	1165 (56.4%)
Employment	
Working	1220 (59%)
Studying	148 (7.2%)
Unemployed/disability	661 (32%)
Pregnancy loss	
No loss	1312 (80%)
Previous miscarriage, termination or stillbirth	328 (20%)
Medical conditions in pregnancy	
Type 1 diabetes	12 (0.6%)
Type 2 diabetes	32 (1.5%)
Hypertension	82 (4%)
High cholesterol	28 (1.4%)

### Gestational weight gain

One third (34.2%; *n* = 692) of women in the study gained weight in excess of the IOM recommendations by 21 weeks and six days. Of the women who were in the normal weight range, 29.7% (*n* = 365) had an excessive rate of weight gain, compared with 43.1% (*n* = 184) of overweight and 45.8% (*n* = 109) of obese women. The distribution of weekly weight gain per BMI group is demonstrated in Table 3. Compared to women with a healthy pre-pregnancy BMI, overweight (odds ratio [OR] 1.79; 95% confidence interval [95% CI] 1.42–2.25; *p <* 0.005) and obese women (OR 2.0, 95% CI, 1.50–2.65; *p <* 0.005) were significantly more likely to gain excess weight.

**Table 3:** Mean and range of early gestational weight gain by pre-pregnancy BMI and the IOM weight gain recommendations [mean (range) in kg] based on self-reported pre-pregnancy BMI

	Underweight (<18.5 kg/m^2^) *n* = 125	Normal weight (BMI 18.5–24.9 kg/m^2^) *n* = 1209	Overweight (BMI 25.0–29.9 kg/m^2^) *n* = 418	Obese (BMI ≥ 30 kg/m^2^) *n* = 229
IOM weekly weight gain recommendation (kg)	0.51 (0.44–0.58)	0.42 (0.35–0.50)	0.28 (0.23–0.33)	0.22 (0.17–0.27)
Weekly weight gain (kg)	0.41 (−1.00–2.60)	0.38 (−2.00–10.00)	0.45 (−8.00–8.00)	0.46 (−2.00–6.33)

*IOM* Institute of Medicine; *BMI* Body mass index; *kg* kilograms

### Predictors of excess gestational weight gain

Univariate analysis of excess GWG (specific to BMI) and sociodemographic predictor variables are shown in Table 4. This demonstrated a significant association of excess weight gain with Middle Eastern/African ethnicity (OR 1.72; 95% CI: 1.28–2.31; *p <* 0.005) and Pacific Islander/Maori background (OR 1.69, 95% CI: 1.07–2.65; *p =* 0.023). Women of Muslim faith were more likely to have excess early GWG compared with women from Christian faith (OR 1.45, 95% CI: 1.09–1.93; *p =* 0.011). Women from a low SES were twice as likely to gain excess weight compared with women from a higher SES (OR 2.07; 95% CI: 1.69–2.54; *p <* 0.005).

**Table 4:** Univariate analysis of the sociodemographic factors of women who gained excess weight in early pregnancy

	Odds ratio	95% CI	*p* value
Age (years)			
16–24 years	1.04	0.81–1.33	0.75
25–34 years	Ref		
≥ 35	0.86	0.68–1.09	0.22
Maternal body mass index (kg/m^2^)			
Normal weight (18.5–24.9)	Ref		
Underweight (< 18.5)	0.85	0.56–1.27	0.42
Overweight (25–29.9)	1.79	1.42–2.25	< 0.005
Obese (> 30)	2.00	1.50–2.65	< 0.005
Relationship status			
Married/de-facto	Ref		
Single/widowed/separated	0.95	0.69–1.30	0.73
Smoking			
Non-smoker	Ref		
Current smoker	0.77	0.50–1.20	0.26
Maternal ethnicity			
Caucasian	Ref		
Asian	0.87	0.71–1.07	0.196
Middle Eastern/Africa	1.72	1.28–2.31	< 0.005
Aboriginal or Torres Strait Islander	1.25	0.60–2.60	0.55
Pacific Islander/Maori	1.69	1.07–2.65	0.023
South American (Latino)	0.70	0.29–1.66	0.41
Maternal religion			
Christian	Ref		
Other (Hindu, Buddhist, Jewish)	0.81	0.61–1.07	0.31
Muslim	1.45	1.09–1.93	0.011
No religion	0.83	0.66–1.03	0.09
Maternal education			
Tertiary	Ref		
High school	1.23	0.99–1.54	0.06
Other (nil, primary)	1.06	0.83–1.35	0.65
Not answered	0.93	0.46–1.92	0.85
Socio-economic status			
High	Ref		
Medium	1.31	0.97–1.76	0.075
Low	2.07	1.69–2.54	< 0.005
Employment			
Employed	Ref		
Unemployed	0.85	0.70–1.02	0.07
Pregnancy loss			
Previous pregnancy loss	Ref		
No pregnancy loss	1.15	0.90–1.49	0.26
Medical conditions in pregnancy			
No diabetes	Ref		
Pre-existing diabetes	0.89	0.46–1.72	0.73
No hypertension	Ref		
Hypertension	0.87	0.54–1.40	0.56

In a multivariate analysis (Table 5), women who were overweight (OR 1.69, 95% CI: 1.33–2.14, *p <* 0.005) or obese (OR 1.64, 95% CI: 1.20–2.24, *p =* 0.002) at booking visit were significantly more likely to have excess GWG compared to normal weight women. Low SES background remained a significant independent factor in predicting excess GWG (OR 1.89; 95% CI: 1.49–2.41; *p <* 0.005). However, there was no significant association seen between ethnicity and excess GWG after adjusting for confounders using multivariate analysis. There were also no significant associations between excessive early GWG with age group, religion, smoking, education or employment.

**Table 5:** Multivariate analysis of maternal sociodemographic factors and excess gestational weight gain

	Odds ratio	95% CI	*p* value
Age (years)			
16–24 years	0.91	0.70–1.19	0.50
25–34 years	Ref		
≥ 35	0.85	0.67–1.09	0.20
Maternal body mass index (kg/m^2^)			
Normal weight (18.5–24.9)	Ref		
Underweight (< 18.5)	0.77	0.50–1.20	0.26
Overweight (25–29.9)	1.69	1.33–2.14	< 0.005
Obese (> 30)	1.64	1.20–2.24	0.002
Smoking			
Non-smoker	Ref		
Current smoker	0.96	0.60–1.53	0.86
Maternal ethnicity			
Caucasian	Ref		
Asian	0.90	0.70–1.15	0.40
Middle Eastern/Africa	1.26	0.89–1.80	0.20
Aboriginal or Torres Strait Islander	1.0	0.47–2.12	0.99
Pacific Islander/Maori	1.13	0.68–1.87	0.65
South American (Latino)	0.62	0.26–1.49	0.28
Maternal religion			
Christian	Ref		
Other (Hindu, Buddhist, Jewish)	0.79	0.57–1.12	0.18
Muslim	1.02	0.72–1.42	0.92
No religion	1.01	0.79–1.27	0.97
Socio-economic status			
High	Ref		
Medium	1.17	0.86–1.59	0.32
Low	1.89	1.49–2.41	< 0.005

### Knowledge regarding GWG

Chi-squared analysis was performed on a relationship between baseline BMI and women’s estimation of weight gain in their pregnancy. Overweight and obese women were significantly more likely to estimate a higher weight than what is recommended for their BMI (χ^2^ = 922.38; *p <* 0.005). The proportion of women who overestimated the amount of weight gain they should gain during pregnancy was, respectively, overweight women 73.5% and obese 85.8%.

Half (51.0%; *n* = 1057) the cohort were unsure about whether excess GWG would affect outcomes for their baby; 10.8% (*n* = 223) did not believe that excess GWG would affect the weight of the baby and 13.7% (*n* = 283) of women did not believe that excess GWG would affect longer term outcomes for their baby. Women who gained weight above the IOM recommendations were more likely to believe that excessive GWG in pregnancy would not have any adverse future effect on health outcomes of their baby (χ^2^ = 6.949; *p <* 0.05) or the weight of the baby (χ^2^ = 16.54; *p <* 0.005) (Table 6).

**Table 6:** Excess gestational weight gain and maternal understanding of the effect on health and weight of the baby

	No excess GWG^a^ (%)	Excess GWG (%)	*p* value
Will excess gestational weight gain affect the weight of the baby?			
Yes	71.7	28.3	< 0.005
No	59.6	40.4	< 0.005
Will excess gestational weight gain affect health outcomes for my baby?			
Yes	72.5	27.5	< 0.005
No	63.3	36.7	< 0.005

^a^GWG: gestational weight gain

## Discussion

This is the largest Australian survey examining health and sociodemographic factors associated with early GWG and women’s understanding of the importance of healthy GWG in pregnancy. We found that in the first half of their pregnancy, more than one third of the cohort (32.4%) gained gestational weight outside the IOM recommendations for their BMI category and the overweight and obese women were more likely to overestimate the appropriate GWG for their baseline BMI. The most significant predictors of excess early GWG were pre-pregnancy BMI and SES status. It is of concern that only half the cohort (51.0%) understood the potential impact of excess GWG on their baby and furthermore, the women who gained the most weight outside the IOM guideline were less likely to believe that excess GWG would impact negatively on their baby. These findings make it clear that education around GWG is required to advise women about what are the expected healthy GWG parameters for their BMI.

Our findings support other studies that have reported women who are overweight and obese women are more likely to gain excess weight as per the IOM guidelines for their BMI category [2, 18, 19]. Recommendations regarding appropriate GWG are particularly relevant to overweight and obese women who are already at risk of complications for their pregnancy and offspring due to their pre-pregnancy weight. Any additional risks that accompany excessive GWG may further complicate their pregnancy, their future health and the health of their child. There are limited data on how many women are gaining excessive weight in early pregnancy in Australia. Only one Australian study (*n* = 664) has examined early gestational weight gain, in the context of overall weight gain in pregnancy. This study reported that by 16 weeks of pregnancy, 8% of women had already reached their recommended weight for the total pregnancy, and 2% had exceeded their total weight gain recommended for the pregnancy. [20] The authors also noted that total gestational weight gain correlated with weight gain in early pregnancy. The LIMIT trial found that delivery of advice about diet, exercise and behavioural changes, to women who are already overweight or obese during pregnancy did not reduce the primary outcome (large for gestational age), nor did it influence GWG despite recruiting between 10 and 20 weeks gestation (intervention 9.39 ± 5.74 kg; standard care 9.44 ± 5.77 kg; *p* = 0.89) [21].

Even though pre-pregnancy BMI is a significant predictor of early excess GWG in our study, up to a third of normal weight women also gained weight in excess of that recommended by the IOM. This is of concern because even women with a healthy weight are at risk, because early excess GWG significantly increases the risk of gestational diabetes, particularly amongst normal weight women [6, 22].

We found women from lower SES backgrounds were more likely to gain weight in early pregnancy in excess of the IOM recommendations. This finding aligns with previous studies which have consistently shown an association between lower SES background and excess GWG [23, 24]. Women who are socio-economically disadvantaged are likely to have greater psychosocial risks such as stress, depression, poor social supports and unplanned pregnancy, which may contribute to poor dietary choices and minimal physical activity [25]. Furthermore, women from higher SES areas tend to be more proactive with seeking information regarding weight gain recommendations, healthy eating and exercise during pregnancy [25].

Our cohort of overweight and obese women was more likely to overestimate their appropriate GWG suggesting that there is a lack of knowledge regarding healthy GWG by women, especially those most at risk – the overweight and the obese. Our findings validate findings from a smaller Australian study (*n* = 364) that examined pregnant women’s knowledge of GWG expectations and their awareness of complications associated with obesity and excess GWG [26]. A second small Australian study recruited pregnant women at 16–18 weeks gestations (*n* = 166), comparing GWG expectations over reported GWG. A significant risk factor for excess GWG was mothers overestimating the minimum amount of GWG needed [15]. Our study differed from these; it was confined to early pregnancy, had a much larger sample size and included women from diverse ethnic and cultural backgrounds.

We have demonstrated the paucity of knowledge by pregnant women regarding the importance of optimal GWG. Preconception care has been shown to be effective with reducing the amount of gain pregnant women gain up until their first booking visit at hospital [27, 28]. Our results call for counselling and discussion regarding both expectations about optimal GWG and consequences of excess GWG. The Cochrane Review of directed preconception health programs and interventions for improving pregnancy outcomes for women who are overweight or obese was unable to include any studies for meta-analysis [29]. These conversations should be initiated preconception or at the very least in early pregnancy with their healthcare practitioner. Despite this being good practice, international studies suggest that few women receive such advice [30–32]. Research exploring reasons for this lack of counselling from healthcare professionals [30, 31] has found the reasons to be limited awareness of current pregnancy weight gain guidelines, belief that such counselling is ineffective, or awkwardness about raising the issue of weight and weight gain with women. If healthcare providers do counsel women and outline expected GWG recommendations, encouragingly there is an increased likelihood of appropriate GWG, particularly when additionally offered lifestyle coaching [33]. The timing of an intervention or patient education is however important. Our study shows women are already gaining excessive weight in early pregnancy and remain at risk of adverse outcomes independent of total GWG and pre-pregnancy BMI.

Our finding that Middle Eastern and Pacific Islander/Maori women were more likely to have excess GWG compared with other ethnic backgrounds was not sustained in the multivariate analysis but warrants further investigation as the evidence is inconclusive around ethnicity as a risk factor for excess GWG [34–38]. Research from New Zealand in the early pregnancy period has reported that Pacific Islander and Maori women women’s knowledge of their BMI and recommended GWG to be inaccurate, irrespective of BMI [39]. There are only a few studies investigating cultural background and GWG and they are commonly from the United States comparing racial differences between Hispanic, white and black women [40, 41]. The recent series in the Lancet on maternal obesity has called for more ethnic and culturally specific research [42]. Cultural background may be an important determinant for GWG, and due to increasing globalisation and migration, it is difficult to ascertain whether any relationship is due to cultural background per se or the result of assimilation into a new environment and culture. Furthermore, there are no current ethnic specific guidelines with regards to healthy GWG. A recent study from Singapore showed that there may be a variation in terms of weight gain recommendations and adverse outcomes specific to those of an Asian ethnicity [43]. Further research is needed to examine whether ethnic specific recommendations for GWG need to be developed and whether there are any specific cultural beliefs that may influence GWG for different ethnic groups.

The strengths of this study is the large number of participants, the inclusion of participants from a number of hospitals (both secondary and tertiary) and the diverse ethnic and cultural backgrounds making these results generalisable to the diverse multicultural Australian population [44]. The inclusion of a multivariable regression analysis assesses a significant association between obese, low SES and excess early gestational weight gain.

A limitation of this study was the use of self-reported pre-pregnancy weight and height, although this has been shown to have adequate correlation with measured height and weight [45]. These results may not be generalisable to other perinatal centres with a distribution of maternal ethnic representation in Australia. There are no current ethnic specific guidelines with respect to ideal weight gain in pregnancy. We could not be certain of specifically when women gained significant weight, whether it be in their first trimester or second trimester therefore we assumed that women gained up to 2 kg in their first trimester and then the weight beyond that can be calculated to be their weekly weight gain.

## Conclusion

Women are gaining weight in early pregnancy outside the recommendations of the IOM, which have been accepted in Australia as the guideline for healthcare professionals to use when counselling women. The women at particular risk of excess early GWG are those who are overweight and obese and women residing in lower SES areas. These women need to be targeted for appropriate counselling preconception or in early pregnancy. This study also suggests that their remains a lack of understanding of healthy weight gain recommendations in early pregnancy, where weight gain has a significant impact on short-term pregnancy outcomes and also long-term consequences for both the mother and child. Given the significant adverse outcomes associated with excess GWG in early pregnancy, preconception or early pregnancy counselling with respect to GWG and intervention research regarding best approach remains a public health priority.

## Additional file

Additional file 1: Table S1. English Questionnaire V2.0. Pregnancy intention and body mass index in women attending clinics. (DOCX 523 kb)

## Abbreviations

95% CI: 95% confidence interval
BMI: body mass index
GWG: gestational weight gain
IOM: Institute of Medicine
OR: odds ratio
RPAH: Royal Prince Alfred Hospital
SES: socio-economic status
SPSS: Statistical Package for the Social Sciences
