# Supplementary material for: Prevalence and predictors of early gestational weight gain associated with obesity risk in a diverse Australian antenatal population: a cross-sectional study
Source: BMC Pregnancy Childbirth. 2017 Sep 7;17:296. doi: 10.1186/s12884-017-1482-6 (PMC5590236; doi:10.1186/s12884-017-1482-6)
Supplement: Additional file 1: Table S1. — English Questionnaire V2.0. Pregnancy intention and body mass index in women attending clinics. (DOCX 523 kb) [file 12884_2017_1482_MOESM1_ESM.docx]

| 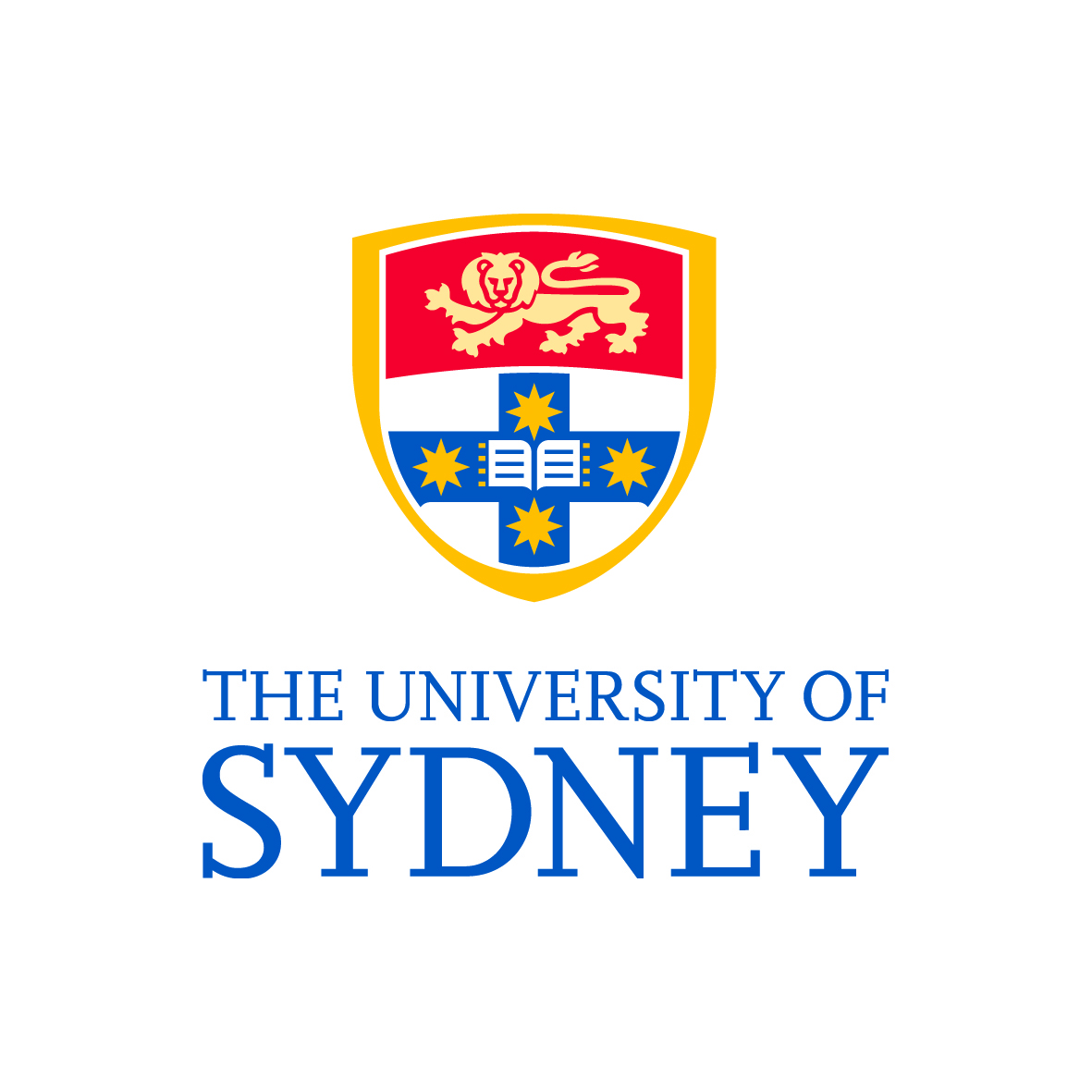 |  |
| --- | --- |

**Pregnancy intention, GWG and body mass index in women**

Thank you for participating in this study. All your responses are confidential and there will be no information stored to identify you. You are taking part in this study because we are interested in how women plan their pregnancies.

**Do you wish to participate in this study? (Please tick the box that applies)**

Yes 🡪Thank you for your participation. Please read on.

No 🡪Thank you for your time. Please return this form to the box provided.

| **Pregnancy intention**  **1. Regarding contraceptive use, at the time of getting pregnant, did you:** | | | |
| --- | --- | --- | --- |
| a. Always use contraception | | |  |
| b. Inconsistently use contraception | | |  |
| c. Not use contraception | | |  |
|  | | | |
| **2. In terms of becoming a mother, is this:** | | | |
| a. The wrong time | | |  |
| b. An OK time, but not quite right | | |  |
| c. The right time | | |  |
|  | | | |
| **3. Just before getting pregnant, did you:** | | | |
| a. Not intend to become pregnant | | |  |
| b. Did not mind either way | | |  |
| c. Intend to get pregnant | | |  |
|  | | | |
| **4. Just before getting pregnant, did you:** | | | |
| a. Not want a baby | | |  |
| b. Have mixed feelings about having a baby | | |  |
| c. Want a baby | | |  |
|  | | | |
| **5. Before getting pregnant, did you:** | | | |
| a. Never discussed children | | |  |
| b. Discuss children, but had no firm agreement | | |  |
| c. Agreed to the pregnancy with your partner | | |  |
|  | | | |
| **6. Before getting pregnant, did you take any actions to prepare for pregnancy such as taking folic acid supplementation, stopping or reducing smoking, stopping or reducing alcohol intake, or seeking medical advice?** | | | |
| a. No actions |  | | |
| b. Health preparations (1 action) |  | | |
| c. Health preparations ( more than 2 actions) |  | | |
| **7. If you were using contraception when you got pregnant, which one were you using?** | | | |
| a. Condoms |  | | |
| b. Pills |  | | |
| c. Withdrawal |  | | |
| d. Natural methods |  | | |
| e. Implants |  | | |
| f. Injections (Depoprovera) |  | | |
| 9. Diaphragm |  | | |
| h. Intrauterine Device |  | | |
| i. No method |  | | |
|  | | | |
| **Health:** We would like to ask some questions about your health and previous pregnancies | | | |
| **8. How many weeks pregnant are you today?** | | _________ weeks | |
| **9. What was your weight before you fell pregnant?** | | _________ kg | |
| **10. What is your current weight?** | | _________ kg | |
| **11. What is your height?** | | _________ cm | |
| **12. Do you have any of these medical conditions?** | | Diabetes (type 1, type 2)  High blood pressure  High cholesterol | |
| **13. Do you smoke?** | | Yes No | |
| **14. Is this your first pregnancy?**  **Have you had a miscarriage or still birth? If yes**  **Number of children? If yes** | | Yes No ; if no  Number ________ Years/s ________  Number ________ Years/s ________ | |
| **15. How much weight are you expecting to gain during your pregnancy?** | | _________ kg | |
| **16. Do you think this is** | | Too much Too little  Just right Not sure | |
| **17.Do you think the amount of weight you gain will affect**  **a. The birthweight of your baby?**  **b. Any other health outcomes for your baby?** | | Yes No Not sure  Yes No Not sure    If yes, please state ______ ___ | |

| **Demographics:** Finally we would like to ask you some questions about yourself | |
| --- | --- |
| **18. How old are you?** | |
| a. 16-17 | d. 35 -39 |
| b. 18-24 | e. 40-45 |
| c. 25-34 |  |
| **19. What is your current marital status?** | |
| a. Single and never been married |  |
| b. Married |  |
| c. Living with a man/woman as a couple |  |
| d. Widowed |  |
| e. Divorced or separated |  |
|  | |
| **20. What is your current employment situation?** | |
| a. Working full time |  |
| b. Working part time |  |
| c. Studying fulltime |  |
| d. Studying part time |  |
| e. Unemployed |  |
| f. On a disability pension |  |
|  | |
| **21. What is the highest qualification you have attained?** | |
| a. Degree level qualification |  |
| b. Diploma |  |
| c. Higher School Certificate |  |
| d. School Certificate |  |
| e. Foreign qualification |  |
| f. Other (please specify) |  |
|  | |
| **22. What is your country of birth and ethnicity?** | Please state__________________ |
| **23. What religion do you belong to?** | Please state__________________ |
| **24. What is your post code?** | Please state__________________ |
| **Thank you for your participation. Please return this form to the box provided.** | |
